# Impacts of gender and lifestyle on the association between depressive symptoms and cardiovascular disease risk in the UK Biobank

**DOI:** 10.1038/s41598-023-37221-x

**Published:** 2023-07-04

**Authors:** Su Nam Lee, Jae-Seung Yun, Seung-Hyun Ko, Yu-Bae Ahn, Ki-Dong Yoo, Sung-Ho Her, Donggyu Moon, Sang-Hyuk Jung, Hong-Hee Won, Dokyoon Kim

**Affiliations:** 1 Division of Cardiology, Department of Internal Medicine, St. Vincent’s Hospital, The Catholic University of Korea, Seoul, Republic of Korea; 2 Catholic Research Institute for Intractable Cardiovascular Disease (CRID), College of Medicine, The Catholic University of Korea, Seoul, Republic of Korea; 3 Division of Endocrinology and Metabolism, Department of Internal Medicine, St. Vincent’s Hospital, The Catholic University of Korea, Seoul, Republic of Korea; 4 Department of Biostatistics, Epidemiology and Informatics, Perelman School of Medicine, University of Pennsylvania, Philadelphia, PA USA; 5 Samsung Advanced Institute for Health Sciences and Technology (SAIHST), Sungkyunkwan University, Samsung Medical Center, Seoul, Republic of Korea; 6 Institute for Biomedical Informatics, University of Pennsylvania, Philadelphia, PA USA; 7 Division of Endocrinology and Metabolism, Department of Internal Medicine, St. Vincent’s Hospital, The Catholic University of Korea, 93, Jungbu-daero, Paldal-gu, Suwon, Gyunggi-do 16247 Republic of Korea

**Keywords:** Cardiology, Health care, Signs and symptoms

## Abstract

We investigated the effects of gender and lifestyle on the association between frequency of depressive symptoms and CVD risk. The UK Biobank is a national prospective cohort study that recruited 502,505 participants aged 40–69 years between 2006 and 2010. Participants without CVD were classified as having low, moderate, high, or very high frequency of depressive symptoms according to the number of days they felt depressed in a 2-week period. UKBB data include self-reported questionnaires covering lifestyle behaviors such as smoking, physical activity, eating habits, and sleep duration. The primary outcomes included incident CVD including coronary artery disease, ischemic stroke, hemorrhagic stroke, peripheral artery disease, atrial fibrillation/flutter, and heart failure. Cox proportional hazard models were used to evaluate the effects of gender and lifestyle on the association of frequency of depressive symptoms and CVD risk. During a median follow-up of 8.9 years, 27,394 (6.3%) developed CVD. The frequency of depressive symptoms increased the risk of CVD according to low, moderate, high, and very high frequency of depressive symptoms (*P* for trend < 0.001). The adjusted CVD risk was 1.38-fold higher for participants with very high frequency of depressive symptoms compared to those with low frequency of depressive symptoms (HR 1.38, 95% CI 1.24–1.53, *P* < 0.001). The correlation between frequency of depressive symptoms and CVD risk was more remarkable in females than in males. In participants with high or very high frequency of depressive symptoms, the individual lifestyle factors of no current smoking, non-obesity, non-abdominal obesity, regular physical activity, and appropriate sleep respectively was associated with lower CVD risk by 46% (HR 0.54, 95% CI 0.48–0.60, *P* < 0.001), 36% (HR 0.64, 95% CI 0.58–0.70, *P* < 0.001), 31% (HR 0.69, 95% CI 0.62–0.76, *P* < 0.001), 25% (HR 0.75, 95% CI 0.68–0.83, *P* < 0.001), and 22% (HR 0.78, 95% CI 0.71–0.86, *P* < 0.001). In this large prospective cohort study, a higher frequency of depressive symptoms at baseline was significantly associated with increased risk of CVD in the middle-aged population, and this relationship was prominent in women. In the middle-aged population with depressive symptoms, engaging in a healthier lifestyle could prevent CVD risk.

## Introduction

Cardiovascular disease (CVD) is the leading cause of death worldwide, accounting for approximately 32% of mortality, and the number of CVD deaths is expected to gradually increase with the increasing age of the population^1^. However, most CVD can be prevented or its onset delayed by modifying unfavorable lifestyles such as smoking, unhealthy diet, and physical inactivity. It is well known that depression is a risk factor of CVD, as previous studies have demonstrated depression to alter serum cholesterol levels and show a U-shaped relationship with body mass index (BMI)^2–5^. Potential common biological mechanisms shared between depression and cardiovascular morbidities have been proposed, including altered circadian rhythms, abnormal hypothalamic–pituitary–adrenal axis activity, imbalanced neurotransmitters, and inflammation^6–9^. A previous study have shown that these two diseases likely share 24 potential pleiotropic genes and biological pathways^10^.

There is a bidirectional causal relationship between depression and CVD. The prevalence of depression in patients with CVD is significantly high; if mild depression is included, approximately 40% of patients with CVD tend to feel depressive^11, 12^. Moreover, depressive patients engage in unfavorable lifestyle behaviors including smoking, obesity, inactivity, and poor diet, which leads to CVD, which in turn leads to a vicious cycle of depression^13^. Depression is also a strong predictor of mortality and rehospitalization in CVD patients^3, 14, 15^. In addition, several studies have shown that psychosocial factors have a greater impact on CVD in women than in men^16, 17^. The comorbidity of depression and cardiometabolic disorders is twice as frequent in women^18, 19^.

In 2022, the American Heart Association released an advisory that presents an updated construct of cardiovascular health (CVH). The major components of CVH include healthy diet, regular physical activity, avoidance of nicotine, healthy sleep, healthy weight, and healthy levels of blood lipids, blood glucose, and blood pressure, defined as “Life’s essential 8”^20^. However, no studies to date have evaluated the impacts of gender and lifestyle on the association between depression and CVD risk. Therefore, we used the UK Biobank data to examine gender differences on the associations between frequency of depressive symptoms and the risk of CVD. We also investigated the interaction of healthy lifestyle behaviors on these relationships in this large prospective cohort study.

## Method

### Study population

The UK Biobank is a national prospective cohort study in the United Kingdom that recruited 502,505 participants aged 40–69 years between 2006 and 2010. Baseline data in the UK Biobank include anthropometric measurements, self-reported questionnaires, and blood samples. Participants with a prior history of CVD (n = 42,649) including coronary artery disease, ischemic stroke, hemorrhagic stroke, peripheral artery disease, atrial fibrillation/flutter, or heart failure at baseline, and those with missing data on depressive symptoms (n = 21,656) were excluded (Additional file 1 in Supplemental Data).

### Cardiovascular health (lifestyle) behaviors

Lifestyle behaviors, except BMI, were assessed with self-reported questionnaires. For smoking status, participants were categorized as either a current smoker or not a current smoker (Additional file 2 in Supplemental Data). Participants with a BMI ≥ 30 kg/m^2^ were defined as being obese in accordance with the World Health Organization international classification^21^. Regarding physical activity, participants were considered to undertake regular physical activity if they reported moderate activity 5 or more days or vigorous activity 3 or more days per week. Healthy eating habits were defined as eating 4 or more of the 7 commonly eaten food groups according to the recommendations on diet priorities for CVH^22^. Overall, on the basis of these four lifestyle factors (smoking, obesity, physical activity, and eating habits), participants were classified as having an ideal (≥ 3 healthy lifestyle factors), intermediate (2 healthy lifestyle factors), or poor (≤ 1 healthy lifestyle factor) lifestyle^23^. Additionally, healthy sleep was defined as getting between 7 and 9 h of sleep each night^20^, and participants were scored on these 5 of Life’s essential 8 lifestyle factors according to the number of good lifestyle habits they practiced. Metabolic health was assessed using the five factors included in the definition of metabolic syndrome (waist circumference, triglycerides, high-density lipoprotein (HDL) cholesterol, blood pressure, and fasting glucose levels)^24^. The Charlson comorbidity index was obtained using the diagnostic codes for myocardial infarction, HF, PAD, cerebrovascular disease, dementia, chronic pulmonary disease, rheumatic disease, liver disease, diabetes mellitus, hemiplegia/paraplegia, renal disease, malignancy, metastatic tumor, and acquired immunodeficiency syndrome (Additional file 3 in Supplemental Data)^25^. Detailed definitions of other variables are described in a previous paper^26^.

### Ascertainment of depressive symptoms

Depressive symptoms were assessed as the self-reported frequency of depressive mood using the 2-week recall method. Participants answered the following question: “Over the past 2 weeks, how often have you felt down, depressive or hopeless?” (UK Biobank field number: 2050). Depressive symptoms were categorized as low (no depressive mood), moderate (several days of depressive mood), high (depressive mood more than half of the past 2 weeks), or very high (nearly every day) frequency. This 2-week recall method is also used in the patient health questionnaire (PHQ)-2 used to screen for depression^27^. Previous study provided the strong evidence for the validity of the PHQ-2 as a depression screening measure^27^.

### Ascertainment of variables

Participants underwent anthropometric and vital sign measurements, as well as providing blood samples. Medication use was extracted at baseline, inclusive of all medications for diabetes, hypertension, and dyslipidemia. We also extracted data on baseline use of medications for depression such as selective serotonin reuptake inhibitors, serotonin/norepinephrine reuptake inhibitors, tricyclic antidepressants, monoamine oxidase inhibitors, bupropion, mirtazapine, and trazodone. Depression diagnosis was determined using the 10th revision of the International Statistical Classification of Disease (ICD-10) (Additional file 3 in Supplemental Data).

### Cardiovascular outcome

For all participants, follow-up linkage to health-related data is available. The primary outcome was incident CVD including CAD, ischemic stroke, hemorrhagic stroke, PAD, AF, and HF, classified according to the 10th revision of the International Statistical Classification of Disease (ICD-10) (Additional file 3 in Supplemental Data).

Cases of incident CVD were identified based on the first recorded disease occurrence and hospitalization records. All participants consented to death registration and subsequent linking to health-related records. The date and cause of death were obtained from death certificates provided by the National Health Service Central Register in Scotland and the National Health Service Information Center in England and Wales. Mortality data from the UK Biobank dataset was provided until 30 November 2016 for centers in Scotland and 31 January 2018 for centers in England and Wales. Causes of death were classified according to the ICD-10.

### Statistical analysis

Participants’ baseline characteristics are expressed as means and standard deviations and assessed for significance using independent *t-*test or analysis of variance for continuous variables, and as percentages and using chi-square tests for categorical variables. Associations of depressive symptoms and CVD risk (both overall and for specific CVD subtypes) were evaluated using Cox proportional hazard models; we also evaluated the interaction of gender and healthy lifestyle on the association between frequency of depressive symptoms and CVD risk. Hazard ratios (HRs) and 95% confidence intervals (CIs) were calculated. A *P* value < 0.05 was considered statistically significant. To account for potentially confounding clinical covariates and to adjust the established risk factors for CVD, multivariable Cox regression models were adjusted for age, sex, and ethnicity (model 1); the variables in model 1 plus the Townsend deprivation index, income, sleep duration, BMI, smoking, alcohol, physical activity, systolic blood pressure, diastolic blood pressure, glycated hemoglobin, triglyceride, HDL cholesterol, low-density lipoprotein (LDL) cholesterol, estimated glomerular filtration rate, C-reactive protein (CRP), all cancer, chronic kidney disease (CKD), chronic lung disease, and chronic liver disease (model 2); and the variables in model 2 plus medication for diabetes, hypertension, dyslipidemia, or depression (model 3). Excluding those diagnosed with depression or prescribed antidepressants at baseline, additional multivariate Cox regression analysis was performed. Individuals were censored on the date of follow-up loss, end of follow-up (31 January 2018 for England and Wales; 30 November 2016 for Scotland), or death, whichever came first. Individuals with missing data were excluded from each model. All statistical analyses were performed using R (version 3.9.0).

### Ethics approval and consent to participate

All participants provided written informed consent for the use of their data for research purpose. The North West Multi-center Research Ethics Committee approved the UK Biobank study. The present study was conducted under application number 67855 of the UK Biobank resource. All research followed the relevant regulations.

## Results

### Baseline characteristics

Baseline demographic and clinical characteristics of the study population are described in Table 1. The mean age of the total study population was 56.6 ± 8.1 years, and 193,749 (44.2%) of participants were men. Participants who had more frequent depressive symptoms were younger, more likely to be female, more likely to have lower income level, more likely to have comorbidities and unfavorable lifestyle behaviors, and had higher CRP and triglyceride levels.

**Table 1:** Baseline characteristics of the population.

Variables	Total (N = 438,200)	Frequency of depressive symptoms			
		Low (N = 335,743)	Moderate (N = 81,141)	High (N = 13,052)	Very high (N = 8264)
Age (years)	56.6 ± 8.1	57.2 ± 8.0	54.9 ± 8.0	54.4 ± 7.9	54.0 ± 7.7
Male	193,749 (44.2)	155,342 (46.3)	30,291 (37.3)	4938 (37.8)	3178 (38.5)
Systolic blood pressure (mmHg)	139.6 ± 19.7	140.7 ± 19.7	136.5 ± 19.2	136.6 ± 19.1	135.4 ± 19.0
Diastolic blood pressure (mmHg)	82.4 ± 10.7	82.7 ± 10.6	81.6 ± 10.8	82.2 ± 10.8	82.0 ± 11.0
BMI (kg/m^2^)	27.3 ± 4.7	27.2 ± 4.6	27.4 ± 5.1	28.2 ± 5.6	28.6 ± 6.0
Waist circumference (cm)	89.7 ± 13.3	89.7 ± 13.1	89.3 ± 13.7	91.2 ± 14.3	92.3 ± 15.0
Body fat percentage (%)	31.4 ± 8.6	31.1 ± 8.4	32.2 ± 8.8	33.0 ± 9.0	33.3 ± 9.4
Whole body fat mass	24.7 ± 9.5	24.4 ± 9.1	25.3 ± 10.2	26.5 ± 11.0	27.3 ± 11.9
Townsend deprivation index	− 1.3 ± 3.1	− 1.6 ± 2.9	− 1.0 ± 3.2	− 0.1 ± 3.5	0.1 ± 3.6
Income level					
Less than 18,000£	78,669 (20.9)	55,060 (19.1)	16,847 (23.8)	3884 (36.7)	2878 (42.8)
18,000 to 30,999£	94,379 (25.1)	72,403 (25.2)	17,704 (25.1)	2642 (25.0)	1630 (24.3)
31,000 to 51,999£	100,504 (26.8)	78,002 (27.1)	18,828 (26.6)	2374 (22.4)	1300 (19.4)
52,000 to 100,000£	80,410 (21.4)	64,186 (22.3)	14,030 (19.9)	1423 (13.4)	771 (11.5)
Greater than 100,000£	21,689 (5.8)	18,041 (6.3)	3244 (4.6)	265 (2.5)	139 (2.1)
Current smoking	44,990 (10.3)	30,164 (9.0)	10,786 (13.3)	2290 (17.5)	1750 (21.2)
Regular physical activity	305,921 (69.8)	238,628 (71.1)	54,586 (67.3)	8125 (62.3)	4582 (55.4)
Poor eating habits	47,278 (10.8)	36,479 (10.9)	8344 (10.3)	1454 (11.1)	1001 (12.1)
Sleep duration (h)	7.2 ± 1.1	7.2 ± 1.0	7.0 ± 1.2	7.0 ± 1.4	6.9 ± 1.8
Lifestyle habits					
Ideal	229,492 (54.9)	182,568 (56.8)	39,131 (50.6)	5135 (42.9)	2658 (36.2)
Intermediate	140,111 (33.5)	105,369 (32.8)	27,298 (35.3)	4529 (37.9)	2915 (39.7)
Poor	48,599 (11.6)	33,676 (10.5)	10,866 (14.1)	2292 (19.2)	1765 (24.1)
Number of good lifestyles					
0	2155 (0.5)	1448 (0.5)	506 (0.7)	97 (0.9)	104 (1.5)
1	27,807 (6.8)	19,273 (6.1)	6295 (8.3)	1199 (10.5)	1040 (14.7)
2	97,210 (23.8)	71,888 (22.8)	19,588 (26.0)	3411 (29.9)	2323 (32.8)
3	146,220 (35.8)	113,268 (36.0)	26,712 (35.4)	3966 (34.8)	2274 (32.1)
4	104,983 (25.7)	83,990 (26.7)	17,664 (23.4)	2215 (19.4)	1114 (15.7)
5	30,267 (7.4)	24,859 (7.9)	4685 (6.2)	503 (4.4)	220 (3.1)
Metabolic health					
0 component	54,076 (14.5)	39,829 (13.9)	11,659 (16.9)	1643 (14.9)	945 (13.7)
1 component	116,096 (31.1)	90,690 (31.6)	20,722 (30.1)	2908 (26.4)	1776 (25.8)
2 components	101,024 (27.0)	78,754 (27.4)	17,674 (25.7)	2801 (25.5)	1795 (26.1)
3 or more components	102,545 (27.4)	77,690 (27.1)	18,843 (27.3)	3650 (33.2)	2362 (34.3)
HbA1c (%)	5.4 ± 0.6	5.4 ± 0.6	5.4 ± 0.6	5.5 ± 0.7	5.5 ± 0.8
eGFR (mL/min/1.73 m^2^)	79.5 ± 14.2	79.0 ± 14.0	81.0 ± 14.4	81.8 ± 15.2	82.0 ± 15.3
Total cholesterol (mg/dL)	222.7 ± 43.0	223.0 ± 42.9	222.6 ± 42.8	220.7 ± 43.6	221.6 ± 45.3
Triglyceride (mg/dL)	153.5 ± 90.4	152.9 ± 89.2	153.1 ± 91.9	161.5 ± 98.7	167.2 ± 102.0
HDL cholesterol (mg/dL)	56.5 ± 14.7	56.5 ± 14.7	56.8 ± 14.9	55.0 ± 14.6	54.4 ± 14.8
LDL cholesterol (mg/dL)	139.4 ± 32.8	139.6 ± 32.7	139.1 ± 32.9	138.4 ± 33.4	139.1 ± 34.2
C-reactive protein (mg/dL)	2.5 ± 4.3	2.5 ± 4.1	2.7 ± 4.5	3.0 ± 4.7	3.3 ± 5.3
Type 2 diabetes	15,547 (3.8)	11,521 (3.7)	2883 (3.9)	667 (5.6)	476 (6.4)
Dyslipidemia	61,095 (13.9)	47,524 (14.2)	10,292 (12.7)	1978 (15.2)	1301 (15.7)
Hypertension	114,371 (26.1)	86,982 (25.9)	21,004 (25.9)	3865 (29.6)	2520 (30.5)
Chronic lung disease	62,222 (14.2)	44,892 (13.4)	13,278 (16.4)	2347 (18.0)	1705 (20.6)
Chronic kidney disease	5197 (1.2)	3978 (1.2)	914 (1.1)	185 (1.4)	120 (1.5)
Chronic liver disease	4504 (1.0)	3141 (0.9)	998 (1.2)	193 (1.5)	172 (2.1)
Cancer	50,017 (11.4)	38,346 (11.4)	9271 (11.4)	1478 (11.3)	922 (11.2)
Anti-depression medication	26,377 (6.0)	12,410 (3.7)	9333 (11.5)	2249 (17.2)	2385 (28.9)
Charson comorbidity index	0.5 ± 0.9	0.5 ± 0.8	0.5 ± 0.9	0.6 ± 0.9	0.6 ± 1.0

*BMI* body mass index, *HbA1C* glycated hemoglobin, *eGFR* estimated glomerular filtration rate, *HDL* high-density lipoprotein, *LDL* low-density lipoprotein.

### Associations between frequency of depressive symptoms and CVD risk

**Figure 1:**
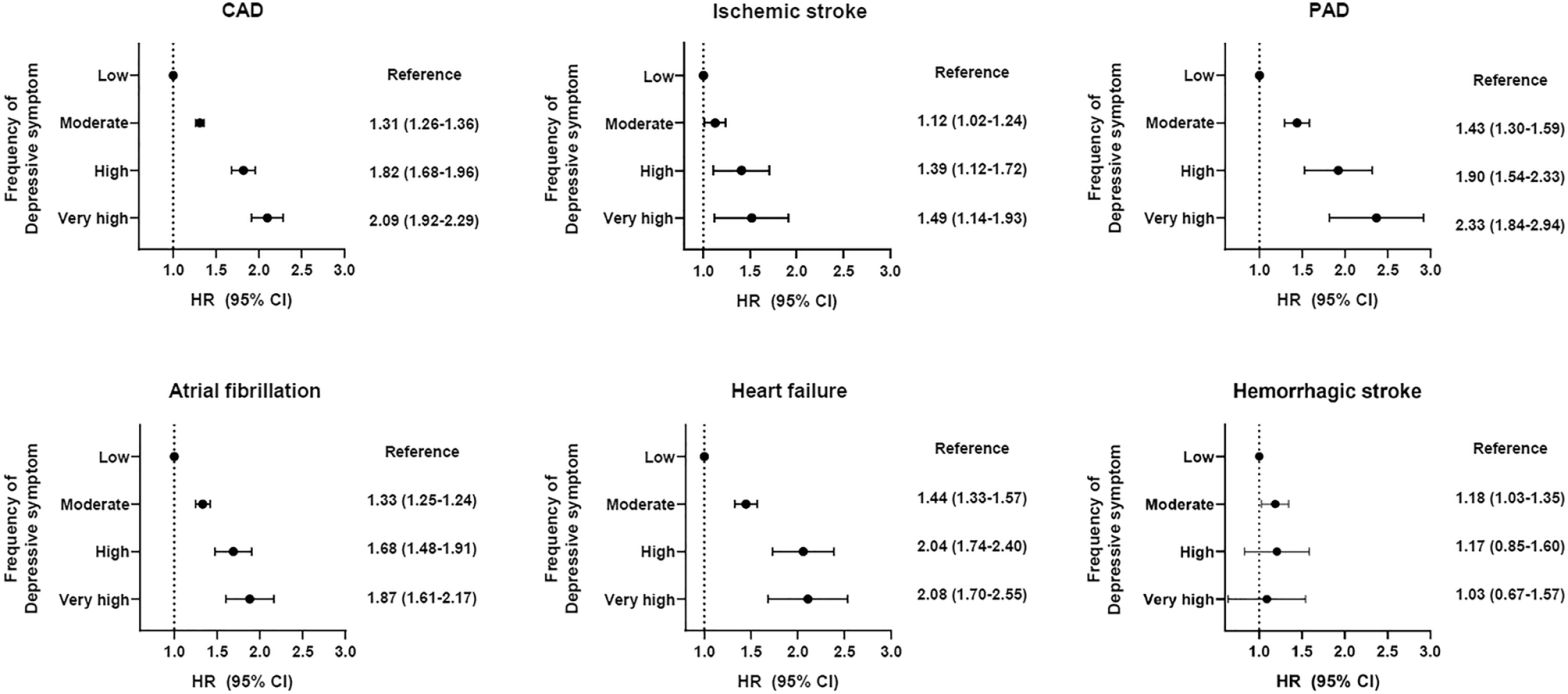
Associations between frequency of depressive symptoms and specific cardiovascular disease risk. *CVD* cardiovascular disease, *CAD* coronary artery disease, *PAD* peripheral artery disease, *AF* atrial fibrillation/flutter, *HF* heart failure. Adjusted for age, sex, and ethnicity.

Participants were followed up with for a median 8.9 years. Among the total population not having any CVD condition at baseline, 27,394 (6.3%) developed CVD. In the Cox regression analysis, frequency of depressive symptoms at baseline was significantly associated with CVD risk. The frequency of depressive symptoms increased the risk of CVD according to low, moderate, high, and very high frequency of depressive symptoms (*P* for trend < 0.001). The adjusted CVD risk was 1.38-fold higher for participants with very high frequency of depressive symptoms compared to those with low frequency of depressive symptoms (HR 1.38, 95% CI 1.24–1.53, *P* < 0.001, Table 2). Among specific CVD outcomes, except for hemorrhagic stroke, similar significant increases of risk were observed in subjects with very high frequency of depressive symptoms: CAD HR 2.09, 95% CI 1.92–2.29, *P* < 0.001; ischemic stroke HR 1.49, 95% CI 1.14–1.93, *P* = 0.003; PAD HR 2.33, 95% CI 1.84–2.94, *P* < 0.001; AF HR 1.87, 95% CI 1.61–2.17, *P* < 0.001; and HF HR 2.08, 95% CI 1.70–2.55, *P* < 0.001 (Fig. 1).

**Table 2:** Associations between frequency of depressive symptoms and cardiovascular disease risk.

Frequency of depressive symptoms	Crude		Model 1		Model 2		Model 3	
	HR (95% CI)	*P*	HR (95% CI)	*P*	HR (95% CI)	*P*	HR (95% CI)	*P*
Low	Ref		Ref		Ref		Ref	
Moderate	1.00 (0.96–1.03)	< 0.001	1.29 (1.25–1.34)	< 0.001	1.20 (1.15–1.25)	< 0.001	1.17 (1.12–1.22)	< 0.001
High	1.37 (1.28–1.47)	< 0.001	1.72 (1.62–1.84)	< 0.001	1.40 (1.28–1.53)	< 0.001	1.35 (1.23–1.47)	< 0.001
Very high	1.50 (1.38–1.63)	< 0.001	2.00 (1.85–2.16)	< 0.001	1.47 (1.32–1.63)	< 0.001	1.38 (1.24–1.53)	< 0.001
*P* for trends		< 0.001		< 0.001		< 0.001		< 0.001

Model1: adjusted for age, sex, and ethnicity. Model 2: model 1 plus adjustment for Townsend deprivation index, income, sleep duration, BMI, smoking, alcohol, physical activity, SBP, DBP, HbA1C, triglyceride, HDL, LDL, eGFR, CRP, all cancer, CKD, chronic lung disease, chronic liver disease. Model 3: model 3 plus medication adjustments for diabetes, hypertension, dyslipidemia, or depression.

### Gender differences on the associations between frequency of depressive symptoms and CVD risk

**Figure 2:**
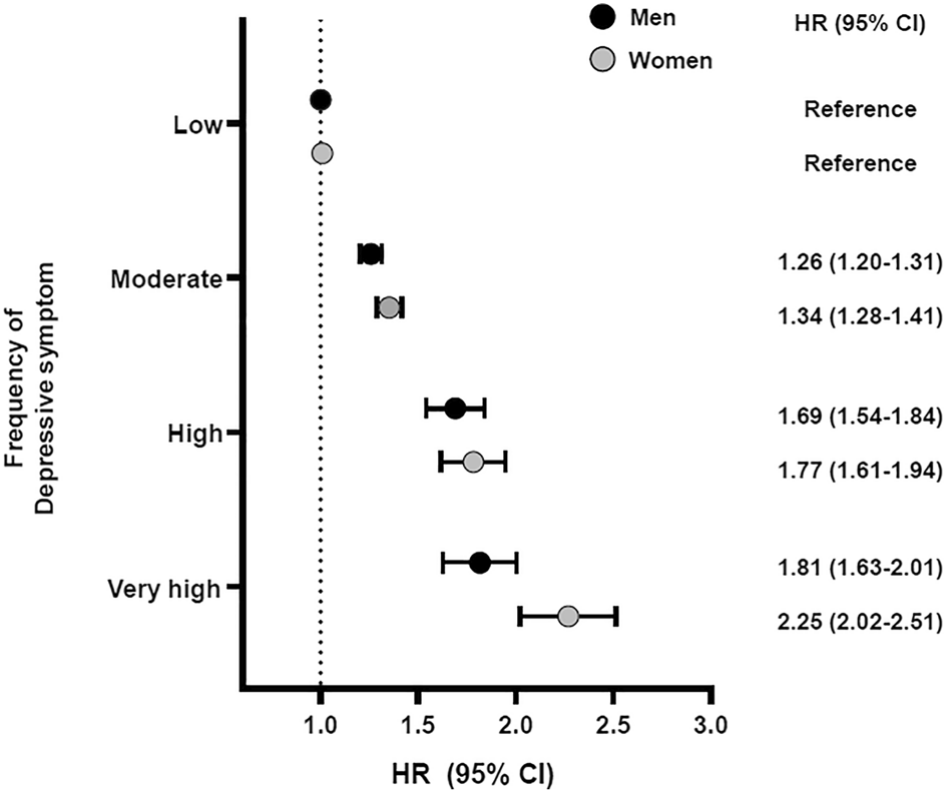
Gender differences on the associations between frequency of depressive symptoms and cardiovascular disease risk.

The relationship of CVD risk to frequency of depressive symptoms was more prominent in female than in male participants (Fig. 2); namely, CVD risk in females was 2.25-fold higher (HR 2.25, 95% CI 2.02–2.51, *P* < 0.001) while that in males was 1.81-fold higher (HR 1.81, 95% CI 1.63–2.01, *P* < 0.001, *P* for interaction 0.012) when comparing participants with very high frequency of depressive symptoms to those with low frequency of depressive symptoms.

### Effect of lifestyles on the associations between frequency of depressive symptoms and CVD risk

**Figure 3:**
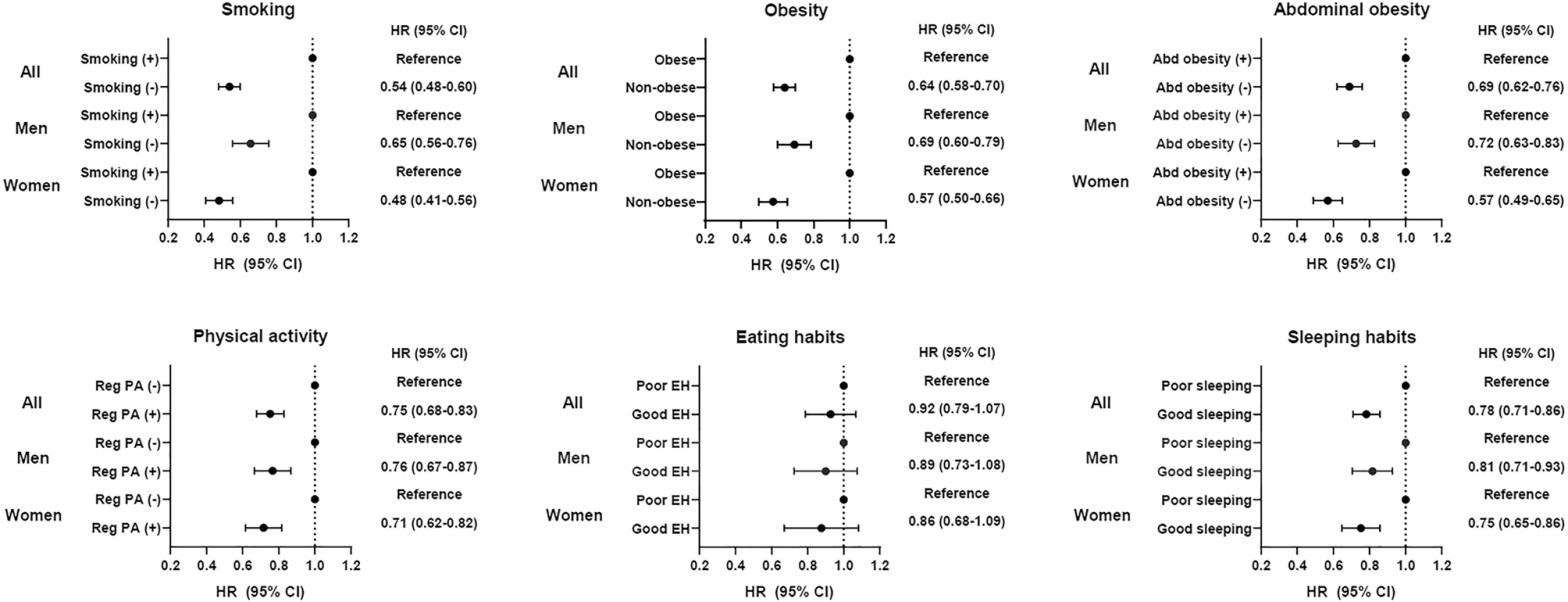
The effect of lifestyle on the associations between frequency of depressive symptoms and cardiovascular disease risk in subjects with greater frequency of depressive symptoms.

In participants with high or very high frequency of depressive symptoms, the individual lifestyle factors of no current smoking, non-obesity, non-abdominal obesity, regular physical activity, and appropriate sleep respectively was associated with lower CVD risk by 46% (HR 0.54, 95% CI 0.48–0.60, *P* < 0.001), 36% (HR 0.64, 95% CI 0.58–0.70, *P* < 0.001), 31% (HR 0.69, 95% CI 0.62–0.76, *P* < 0.001), 25% (HR 0.75, 95% CI 0.68–0.83, *P* < 0.001), and 22% (HR 0.78, 95% CI 0.71–0.86, *P* < 0.001) (Fig. 3). Meanwhile, eating habits did not affect risk of CVD. In relation to gender, several healthy lifestyle factors were found to play significant roles in reducing CVD risk for females more so than for males; these were; no current smoking (HR 0.48, 95% CI 0.41–0.56, *P* < 0.001 in females; HR 0.65, 95% CI 0.56–0.76, *P* < 0.001 in males; *P* for interaction 0.01), non-abdominal obesity (HR 0.57, 95% CI 0.49–0.65, *P* < 0.001 in females; HR 0.72, 95% CI 0.63–0.83, *P* < 0.001 in males; *P* for interaction 0.012), and non-obesity (HR 0.57, 95% CI 0.50–0.66, *P* < 0.001 in females; HR 0.69, 95% CI 0.60–0.79, *P* < 0.001 in males; *P* for interaction 0.049).

The associations between frequency of depressive symptoms and CVD risk were maintained even when excluding those diagnosed with depression or prescribed antidepressants at baseline (Additional file 4 in Supplemental Data); likewise, healthy lifestyle factors except eating habits still was associated with lower CVD risk (Additional file 5 in Supplemental Data).

## Discussion

The principal findings in the present study are as follows: a higher frequency of depressive symptoms at baseline is associated with higher CVD risk in the middle-aged population. Such association was also observed in the specific CVD outcomes of CAD, ischemic stroke, PAD, AF, and HF, but not in hemorrhagic stroke. The relationship between frequency of depressive symptoms and risk of CVD was more remarkable in women than in men. Moreover, in the population with high frequency of depressive symptoms, avoidance of nicotine, healthy weight, regular physical activity, and appropriate sleep duration could reduce CVD risk regardless of gender. To our knowledge, this work is the first national, prospective cohort study to evaluate the effects of gender and lifestyles on the associations between frequency of depressive symptoms and CVD risk.

It is well known that patients with CVD are more likely to have depressive moods, and that patients with depression are at high risk of CVD^13, 28^. Depression doubles the risk of developing CVD, and the occurrence of depression in patients with acute myocardial infarction increases the mortality rate by 3 times compared to those without depression^3, 29^. Depression and CVD have common risk factors, such as high prevalence of chronic diseases (including diabetes, hypertension, dyslipidemia, and CKD) and high levels of CRP and cholesterol^13^. These results were similarly reflected in our findings. Several putative biological mechanisms have been proposed to explain the bidirectional association between depression and CVD, including autonomic nervous system dysregulation^30^; platelet receptor and function change^31^; inflammatory biomarkers such as interleukin-6, tumor necrosis factor-alpha, CRP, or coagulation factors^32^; endothelial dysfunction; and genetic variation of the serotonin transporter^33^. Previous studies have shown that elevated depressive symptoms are independently associated with poor metabolic disturbance, such as increased waist circumference and triglycerides, and with inflammation^34, 35^.

Significant sex differences exist in aspects of CVD, including differences in risk factors, age of onset, symptom presentation, treatment, and outcome^36^. In particular, risk of CVD in women is greatly increased relative to that in men by comorbidities such as diabetes, hypertension, and dyslipidemia; moreover, socioeconomic and psychosocial factors also seem to have greater effect on CVD in women^16, 17, 37^. The comorbidity of depression and cardiometabolic disorders is twice as high in women^18, 19^. Gender differences in the comorbidity of depression and cardiometabolic disorder stem from pathogenic processes that originate in fetal development, involving shared pathophysiology between the brain, vascular system, central nervous system control of the heart and related hormonal, immune, and metabolic physiology^18^. Our findings showed that frequency of depressive symptoms increased CVD risk in women higher than that in men.

The present study showed that the frequency of depressive symptoms at baseline was significantly associated with elevated CVD risk in the middle-aged population. However, some researchers reported that depression in older adults was not associated with increased risk of CVD^38^. Zhang et al. investigated the association between depression duration and subsequent CVD using a territory-wide population cohort^38^. They found that duration of depression was positively associated with CVD only in adults younger than 65 years. A possible explanation might be the difference in inflammation levels according to onset age of depression. A previous clinical epidemiological study suggested that patients with early-onset depression had higher levels of inflammatory biomarkers than those with late-onset depression^39^.

Overall, as the frequency of depressive symptoms increased, risk of CVD also increased. In addition, when analyzing each respective CVD outcome, depressive symptoms were associated with all CVDs except for hemorrhagic stroke; that is not only atherosclerotic CVD (ASCVD) but also non-ASCVD outcomes such as HF and AF were associated with depressive symptoms. Although there have been previous studies showing that depression is associated with risk of stroke, this has mainly concerned ischemic stroke^40, 41^. Our results are consistent with a prior Japanese study that showed depression to significantly increase the risk of total and ischemic stroke, but not hemorrhagic stroke^40^.

Lifestyle interventions are important not only for the prevention and treatment of depression, but also for the prevention of other comorbidities. Moreover, patients with depression are less likely to adhere to medication and lifestyle interventions than those without depression^42, 43^. Recently, the American Heart Association has announced the essential 8 components including lifestyle factors to improve and maintain CVH^20^. However, it has not yet been studied the effect of healthy lifestyle behaviors on the association between frequency of depressive symptoms and CVD risk. Our study found that nicotine avoidance, a healthy weight, regular physical activity, and adequate sleep duration could reduce the risk of CVD in participants reporting greater frequency of depressive symptoms. Meanwhile, eating habits did not affect the CVD risk. Previous studies showed that healthy diet has been inversely associated with CVD risk^44–46^. However, there are complexities in measuring dietary quality and heterogenous definition of healthy diet^47^. In addition, various biologic, social, economic, and psychological factors affect food selection. These findings may explain our results that eating habit did not affect CVD risk in participants reporting greater frequency of depressive symptoms.

Major strengths of the present study were the large sample size of over 430,000 participants and the prospective design with detailed anthropometric, lifestyle, and laboratory results. However, this work also has several limitations. First, this study was conducted with individuals of European ancestry, which limits generalization of the results to other ethnic groups. Second, we carefully adjusted for major confounding factors, but unmeasured confounders might have affected the outcomes. Third, self-reported questionnaires on depressive symptoms are not sufficient to represent depression. Degree of depressive mood was determined based only on a single 2-week recall measure. However, this recall method is also used in the PHQ-2 used to screen for depression and the PHQ-2 has been validated^27^. And, other symptoms of depression such as appetite and fatigue were not measured, only the frequency of depressive mood was recorded. Fourth, frequency of depressive mood and lifestyle behaviors were assessed only at baseline; no follow-up changes were assessed. This may underestimate the association between episodic or time-dependent depressive mood and CVD risk. The present study design does not allow investigation of the potential effects of appropriately treating depression on CVD outcomes. However, previous studies have shown that participants having initially high levels of depressive mood maintained their depressive mood at follow-up^48^. To clarify the effects of lifestyle and depressive mood changes over time on the CVD risk, further prospective study is warranted. Fifth, information on disease history and lifestyle was self-reported, and cause of death was obtained based on diagnostic code, thus measurement errors were unavoidable. Most large-scale epidemiological studies rely on self-reported lifestyle behaviors, but self-reported physical activity has been found to be moderately correlated with objective accelerometer measurements^49^. Finally, the present study was an observation study, and causality cannot be determined.

In conclusion, we found that higher frequency of depressive symptoms at baseline is an independent risk factor of CVD in the middle-aged population. The relationship between frequency of depressive symptoms and risk of CVD was more remarkable in women than in men. In addition, the present study demonstrated the potential benefits of healthy lifestyles, including avoidance of nicotine, healthy body weight, regular physical activity, and appropriate sleep duration, in preventing the CVD risk in the population with depressive symptoms. Clinicians should emphasize the importance of healthy lifestyle for the middle-aged population with depressive symptoms.

## Supplementary Information

Supplementary Information.

## Data Availability

This datasets used and analysed during the current study available from the corresponding author on reasonable request.

## Abbreviations

CVD: Cardiovascular disease
BMI: Body mass index
CVH: Cardiovascular health
CAD: Coronary artery disease
PAD: Peripheral artery disease
AF: Atrial fibrillation/flutter
HF: Heart failure
HR: Hazard ratio
CI: Confidence interval
HDL: High-density lipoprotein
LDL: Low-density lipoprotein
CRP: C-reactive protein
CKD: Chronic kidney disease
ASCVD: Atherosclerotic CVD
