# Supplementary material for: Impacts of gender and lifestyle on the association between depressive symptoms and cardiovascular disease risk in the UK Biobank
Source: Sci Rep. 2023 Jul 4;13:10758. doi: 10.1038/s41598-023-37221-x (PMC10319713; doi:10.1038/s41598-023-37221-x)
Supplement: Supplementary file 1 — Supplementary Information. [file 41598_2023_37221_MOESM1_ESM.docx]

**Supplemental File 1. Flow chart**

**
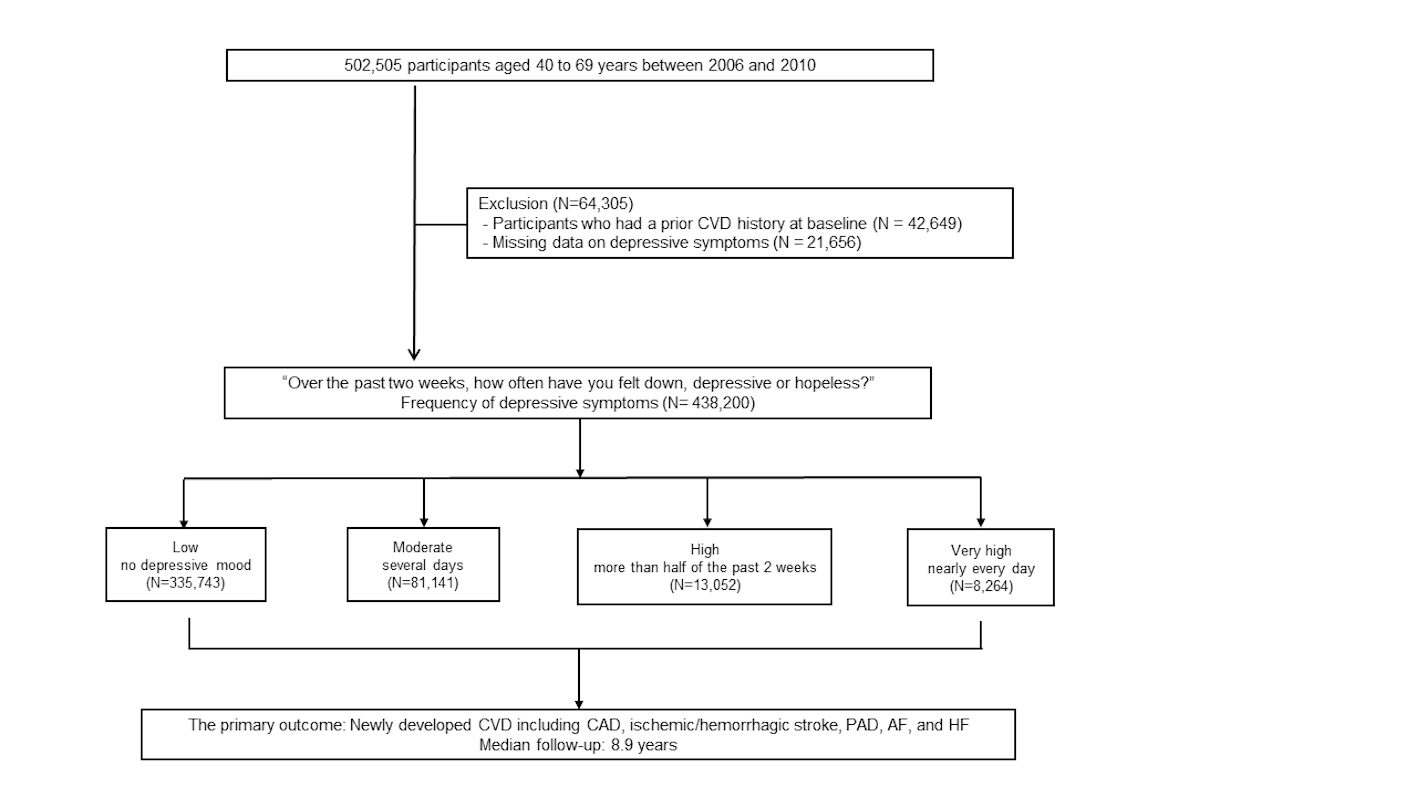
**

**Supplemental File 2. Detailed definitions of lifestyle factors, lifestyle behavior and metabolic health**

| **Lifestyle factors** | **Component** | **Healthy lifestyle or metabolic status** | **Field ID of UK biobank** |
| --- | --- | --- | --- |
| Current smoking | Current smoking at baseline | Absence | 20116 |
| Obesity | BMI at baseline | <30 kg/m^2^ | 21001 |
| Physical activity | Number of days per week of physical activity 10+ minutes | Participating in moderate activity ≥5 days a week or vigorous activity ≥3 days a week | 884 (Moderate physical activity 10+ minutes) 904 (Vigorous physical activity 10+ minutes) |
| Eating habits | At least half of all following diet components was considered as a healthy lifestyle, less than half was considered as an unhealthy lifestyle | | |
|  | Fruit | ≥3 serving/day | 1309 (Fresh fruit) 1319 (Dried fruit) |
|  | Vegetable | ≥3 serving/day | 1289 (Cooked vegetables) 1299 (Salad or raw vegetables) |
|  | Whole grains | ≥3 serving/day | 1438, 1448 (Wholemeal or wholegrain bread) 1458, 1468 (Bran, oat, muesli cereal) |
|  | Fish | ≥2 serving/week | 1329 (Oily fish) 1339 (Non-oily fish) |
|  | Dairy | ≥2.5 serving/week | 1408 (Cheese) 1418 (Milk) |
|  | Refined grains | ≤1.5 serving/week | 1438, 1448 (Wholemeal or wholegrain bread) 1458, 1468 (Bran, oat, muesli cereal) |
|  | Processed meats | ≤1 serving/week | 1349 (Processed meat) 3680 (Age when last ate any kind of meat, 0 if indicated having never eaten meat) |
|  | Unprocessed meats | ≤1.5 serving/week | 1359 (Poultry) 1369 (Beef) 1379 (Lamb) 1389 (Pork) 3680 (Age when last ate any kind of meat, 0 if indicated having never eaten meat) |
|  | Sugar-sweetened beverages | ≤1 serving/week | 6144 (Never eats sugar or foods/drinks containing sugar) |
| Sleep habits | Sleep duration | 7-9 hours/day | 1160 |
| Lifestyle behavior | Favorable | Having at least three healthy lifestyle factors | |
|  | Intermediate | Having two healthy lifestyle factors | |
|  | Unfavorable | Having one or fewer healthy lifestyle factor | |
| Components of metabolic syndrome | Waist circumference | Men <102 cm  Women <88 cm | 48 |
|  | Triglyceride | <1.70 mmol/L | 30870 |
|  | HDL cholesterol | ≥1.03 mmol/L | 30760 |
|  | Glucose  (or having diabetes) | ≥5.6 mmol/L | 30740 |
|  | Systolic blood pressure  or diastolic blood pressure | ≥130 mmHg or  ≥ 85 mmHg | 4080  4079 |

**Supplemental File 3. Detailed definitions of baseline major comorbidities and outcomes**

| **Disease** | **Path** | **Field ID** | **Code** |
| --- | --- | --- | --- |
| Baseline dyslipidemia | Verbal interview | Non-cancer illness, self-report (20002) | High cholesterol (1473) |
|  | First occurrence before enrollment | First reported of disorders of lipoprotein metabolism and other lipidemia (130815, 130816) | E78.x |
|  | Medication | Medication for cholesterol, blood pressure or diabetes (6177) | Cholesterol lowering medication |
| Baseline hypertension | Verbal interview | Non-cancer illness, self-report (20002) | 1065, 1072 |
|  | Touchscreen | Vascular/heart problems diagnosed by doctor (6150) | High blood pressure |
|  | First occurrence before enrollment | First reported of essential hypertension (131286, 131287) | I10.x |
|  |  | First reported of hypertensive heart disease (131288, 131289) | I11.x |
|  |  | First reported of hypertensive renal disease (131290, 131291) | I12.x |
|  |  | First reported of hypertensive heart and renal disease (131292, 131293) | I13.x |
|  |  | First reported of secondary hypertension (131294, 131295) | I15.x |
|  | Medication | Medication for cholesterol, blood pressure or diabetes (6177) | Blood pressure medication |
| Baseline type 2 diabetes mellitus | Verbal interview | Non-cancer illness, self-report (20002) | Diabetes (1220) |
|  |  |  | Type 2 diabetes (1223) |
|  | Touchscreen | Diabetes diagnosed by doctor (2443) | Yes |
|  | First occurrence before enrollment | First reported of non-insulin-dependent diabetes mellitus (130708, 130709) | E11.x |
|  |  | First reported of unspecified diabetes mellitus (130714, 130715) | E14.x |
|  | Medication | Treatment/medication code (20003) | Insulin (1140883066) |
|  |  |  | Metformin (1140884600, 1141189090) |
|  |  |  | Sulfonylurea (1141152590, 1140874744, 1140874718, 1141156984) |
|  |  |  | Acarbose (1140868902) |
|  |  |  | Thiazolidinedione (1141171646) |
|  |  |  | Meglitinide (1141168660, 1141173882) |
|  | HbA1c at baseline | Glycated hemoglobin (HbA1c) (30750) | ≥6.5% |
|  | Verbal interview for exclusion type 1 diabetes | Non-cancer illness, self-report (20002) | Type 1 diabetes (1222) |
|  | First occurrence for exclusion type 1 diabetes | First reported of insulin-dependent diabetes mellitus (130706, 130707) | E10.x |
| Baseline coronary artery disease | Verbal interview | Non-cancer illness, self-report (20002) | 1074 (angina) |
|  |  |  | 1075 (heart attack/myocardial infarction) |
|  | Touchscreen | Vascular/heart problems diagnosed by doctor (6150) | Heart attack, angina |
|  | First occurrence before enrollment | First reported of angina pectoris (131296, 131297) | I20.x |
|  |  | First reported of acute myocardial infarction (131298, 131299) | I21.x |
|  |  | First reported of subsequent myocardial infarction (131300, 131301) | I22.x |
|  |  | First reported of certain current complications following acute myocardial infarction (131302, 131303) | I23.x |
|  |  | First reported of other acute ischemic heart diseases (131304, 131305) | I24.x |
|  |  | First reported of chronic ischemic heart disease (131306, 131307) | I25.x |
| Baseline peripheral artery disease | Verbal interview | Non-cancer illness, self-report (20002) | Peripheral vascular disease (1067) |
|  | Hospital inpatient data | Summary Diagnosis (ICD10, 41270) | I70.0, I70.00, I70.01, I70.2, I70.21, I70.8, I70.80, I70.9, I70.90, I73.8, I73.9 |
|  |  | Summary Diagnosis (ICD9, 41271) | 4400, 4402, 4438, 4439 |
| Baseline heart failure | Verbal interview | Non-cancer illness, self-report (20002) | Heart failure (1076, 1079) |
|  | Hospital inpatient data | Summary Diagnosis (ICD10, 41270) | I50, I50.0, I50.1, I50.9 |
|  |  | Summary Diagnosis (ICD9, 41271) | 4254, 4280, 4281, 4289 |
| Baseline heart arrhythmia | Verbal interview | Non-cancer illness, self-report (20002) | Heart arrhythmia (1094) |
|  | First occurrence before enrollment | First reported of atrial fibrillation/flutter (131350, 131351) | I48.x |
|  |  | First reported of other cardiac arrhythmias (131352, 131353) | I49.x |
| Baseline ischemic stroke | Verbal interview | Non-cancer illness, self-report (20002) | Stroke (1081,1082,1583) |
|  | First occurrence before enrollment | First reported of cerebral infarction (131366, 131367) | I63.x |
| Baseline hemorrhagic stroke | Verbal interview | Non-cancer illness, self-report (20002) | Stroke (1081) |
|  | First occurrence before enrollment | First reported of subarachnoid hemorrhage (131360, 131361) | I60.x |
|  |  | First reported of intracerebral hemorrhage (131362, 131363) | I61.x |
|  |  | First reported of other nontraumatic intracranial hemorrhage (131364, 131365) | I62.x |
| Baseline depression | Hospital inpatient data | Summary Diagnosis (ICD10, 41270) | F32.x, F33.x |
|  | Medication | Medication for depression (6177) | Antidepressant medication |
| Baseline chronic lung disease | Verbal interview | Non-cancer illness, self-report (20002) | Asthma (1111) |
|  |  |  | Chronic obstructive airway disease/COPD (1112) |
|  |  |  | Emphysema/chronic bronchitis (1113) |
|  |  |  | Bronchiectasis (1114) |
|  |  |  | Interstitial lung disease (1115) |
|  | First occurrence before enrollment | First reported of bronchitis, not specified as acute or chronic (131484, 131485) | J40.x |
|  |  | First reported of simple and mucopurulent chronic bronchitis (131486, 131487) | J41.x |
|  |  | First reported of unspecified chronic bronchitis (131488, 131489) | J42.x |
|  |  | First reported of emphysema (131490, 131491) | J43.x |
|  |  | First reported of other chronic obstructive pulmonary disease (131492, 131493) | J44.x |
|  |  | First reported of asthma (131494, 131495) | J45.x |
|  |  | First reported of status asthmaticus (131496, 131497) | J46.x |
|  |  | First reported of bronchiectasis (131498, 131499) | J47.x |
|  |  | First reported of coalworker's pneumoconiosis (131500, 131501) | J60.x |
|  |  | First reported of pneumoconiosis due to asbestos and other mineral fibers (131502, 131503) | J61.x |
|  |  | First reported of pneumoconiosis due to dust containing silica (131504, 131505) | J62.x |
|  |  | First reported of pneumoconiosis due to other inorganic dusts (131506, 131507) | J63.x |
|  |  | First reported of unspecified pneumoconiosis (131508, 131509) | J64.x |
|  |  | First reported of airway disease due to specific organic dust (131512, 131513) | J66.x |
|  |  | First reported of hypersensitivity pneumonitis due to organic dust (131514, 131515) | J67.x |
| Baseline chronic liver disease | Verbal interview | Non-cancer illness, self-report (20002) | Hepatitis (1155) |
|  |  |  | Infective/viral hepatitis (1156) |
|  |  |  | Non-infective hepatitis (1157) |
|  |  |  | Liver failure/cirrhosis (1158) |
|  | First occurrence before enrollment | First reported of chronic viral hepatitis (130200, 130201) | B18.x |
|  |  | First reported of esophageal varices (131406, 131407) | I85.x |
|  |  | First reported of alcoholic liver disease (131658, 131659) | K70.x |
|  |  | First reported of toxic liver disease (131660, 131661) | K71.x |
|  |  | First reported of hepatic failure, not elsewhere classified (131662, 131663) | K72.x |
|  |  | First reported of chronic hepatitis, not elsewhere classified (131664, 131665) | K73.x |
|  |  | First reported of other diseases of liver (131670, 131671) | K76.x |
| Baseline chronic kidney disease | Verbal interview | Non-cancer illness, self-report (20002) | Renal/kidney failure (1192) |
|  |  |  | Renal failure requiring dialysis (1193) |
|  |  |  | Renal failure not requiring dialysis (1194) |
|  | First occurrence before enrollment | First reported of chronic renal failure (132030, 132031) | N18.x |
|  |  | First reported of unspecified renal failure (132032, 132033) | N19.x |
| Baseline cancer | Verbal interview | Cancer, self-report (20001) | 1001-1012, 1015-1048, 1050-1053, 1055,1056, 1058-1068, 1070-1082, 1084-1088 |
|  | First occurrence before enrollment | Cancer register (40006) | C00-C97 |
| Coronary artery disease outcome | First occurrence before enrollment | First reported of angina pectoris (131296, 131297) | I20.x |
|  |  | First reported of acute myocardial infarction (131298, 131299) | I21.x |
|  |  | First reported of subsequent myocardial infarction (131300, 131301) | I22.x |
|  |  | First reported of certain current complications following acute myocardial infarction (131302, 131303) | I23.x |
|  |  | First reported of other acute ischemic heart diseases (131304, 131305) | I24.x |
|  |  | First reported of chronic ischemic heart disease (131306, 131307) | I25.x |
| Peripheral artery disease outcome | Hospital inpatient data | Summary Diagnosis (ICD10, 41270) | I70.0, I70.00, I70.01, I70.2, I70.21, I70.8, I70.80, I70.9, I70.90, I73.8, I73.9 |
|  |  | Summary Diagnosis (ICD9, 41271) | 4400, 4402, 4438, 4439 |
| Heart failure outcome | Hospital inpatient data | Summary Diagnosis (ICD10, 41270) | I50, I50.0, I50.1, I50.9 |
|  |  | Summary Diagnosis (ICD9, 41271) | 4254, 4280, 4281, 4289 |
| Atrial fibrillation/atrial flutter outcome | First occurrence before enrollment | First reported of atrial fibrillation/flutter (131350, 131351) | I48.x |
| Ischemic stroke | First occurrence before enrollment | First reported of cerebral infarction (131366, 131367) | I63.x |
| Hemorrhagic stroke | First occurrence before enrollment | First reported of subarachnoid hemorrhage (131360, 131361) | I60.x |
|  |  | First reported of intracerebral hemorrhage (131362, 131363) | I61.x |
|  |  | First reported of other nontraumatic intracranial hemorrhage (131364, 131365) | I62.x |

**Supplementary File 4. Associations between frequency of** **depressive symptoms and cardiovascular disease risk in subjects without diagnosed depression or antidepressants**

| Frequency of depressive symptoms | Crude | | Model 1 | | Model 2 | | Model 3 | |
| --- | --- | --- | --- | --- | --- | --- | --- | --- |
|  | HR (95% CI) | *P* | HR (95% CI) | *P* | HR (95% CI) | *P* | HR (95% CI) | *P* |
| Low | Ref |  | Ref |  | Ref |  | Ref |  |
| Moderate | 1.02 (0.99-1.06) | 0.225 | 1.30 (1.25-1.34) | <0.001 | 1.20 (1.15-1.25) | <0.001 | 1.17 (1.12-1.22) | <0.001 |
| high | 1.31 (1.23-1.41) | <0.001 | 1.71 (1.59-1.83) | <0.001 | 1.39 (1.27-1.53) | <0.001 | 1.33 (1.21-1.46) | <0.001 |
| Very high | 1.51 (1.39-1.63) | <0.001 | 2.02 (1.87-2.19) | <0.001 | 1.51 (1.35-1.69) | <0.001 | 1.41 (1.26-1.58) | <0.001 |

Model1: adjusted for age, sex, and ethnicity. Model 2: model 1 plus adjustment for Townsend deprivation index, income, sleep duration, BMI, smoking, alcohol, physical activity, SBP, DBP, HbA1C, triglyceride, HDL, LDL, eGFR, CRP, all cancer, CKD, chronic lung disease, chronic liver disease. Model 3: model 3 plus medication adjustments for diabetes, hypertension, dyslipidemia, or depression.

**Supplementary File 5. The effect of lifestyle on the associations between frequency of** **depressive symptoms and cardiovascular disease risk in subjects with greater frequency of depressive symptoms without diagnosed depression or antidepressants**

| Lifestyle factor | All | | Male | | Female | | *P* for interaction |
| --- | --- | --- | --- | --- | --- | --- | --- |
|  | HR (95% CI) | *P* | HR (95% CI) | *P* | HR (95% CI) | *P* |  |
| Smoking (+) | Ref |  | Ref |  | Ref |  | 0.060 |
| Smoking (-) | 0.54 (0.48-0.60) | <0.001 | 0.63 (0.53-0.74) | <0.001 | 0.49 (0.41-0.58) | <0.001 |  |
| Obesity (+) | Ref |  | Ref |  | Ref |  | 0.048 |
| Obesity (-) | 0.67 (0.61-0.75) | <0.001 | 0.72 (0.62-0.83) | <0.001 | 0.56 (0.48-0.65) | <0.001 |  |
| Abdominal obesity (+) | Ref |  | Ref |  | Ref |  | 0.013 |
| Abdominal obesity (-) | 0.69 (0.62-0.76) | <0.001 | 0.72 (0.63-0.83) | <0.001 | 0.57 (0.49-0.65) | <0.001 |  |
| Regular PA (-) | Ref |  | Ref |  | Ref |  | 0.047 |
| Regular PA (+) | 0.60 (0.55-0.67) | <0.001 | 0.66 (0.57-0.77) | <0.001 | 0.55 (0.47-0.63) | <0.001 |  |
| Unhealthy eating habit | Ref |  | Ref |  | Ref |  | 0.765 |
| Healthy eating habit | 0.89 (0.75-1.04) | 0.146 | 0.82 (0.65-1.02) | 0.077 | 0.87 (0.68-1.11) | 0.261 |  |
| Unhealthy sleep | Ref |  | Ref |  | Ref |  | 0.259 |
| Healthy sleep | 0.77 (0.69-0.85) | <0.001 | 0.81 (0.70-0.94) | 0.005 | 0.73 (0.63-0.85) | <0.001 |  |

Adjusted for age, sex, and ethnicity. PA, physical activity.

**Supplementary File 6. Directed acyclic graph of the association between depressive symptoms and CVD**

**
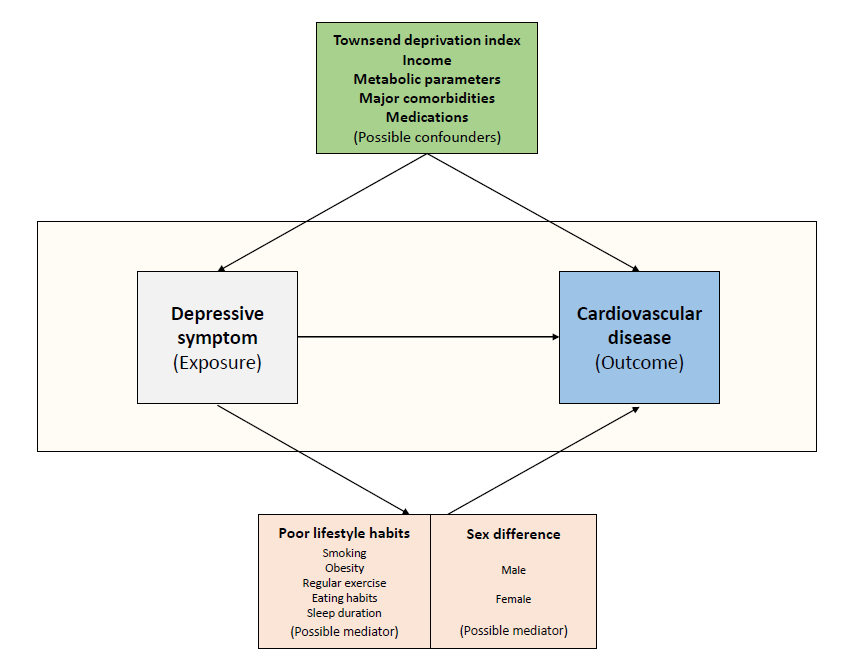
**
